# Comparison of Different Methods for Spongin-like Collagen Extraction from Marine Sponges (*Chondrilla caribensis* and *Aplysina fulva*): Physicochemical Properties and In Vitro Biological Analysis

**DOI:** 10.3390/membranes11070522

**Published:** 2021-07-12

**Authors:** Tiago A. T. Araújo, Amanda de Souza, Alan F. Santana, Anna Rafaela C. Braga, Márcio R. Custódio, Fábio R. Simões, Gabriela M. Araújo, Antônio Miranda, Flávio Alves, Renata N. Granito, Na Yu, Ana Claudia M. Renno

**Affiliations:** 1 Department of Biosciences, Universidade Federal de São Paulo (UNIFESP), Santos 11015-220, SP, Brazil; amanda_desouza@outlook.com (A.d.S.); alandefranca@hotmail.com (A.F.S.); anna.braga@unifesp.br (A.R.C.B.); re_neves@yahoo.com.br (R.N.G.); acmr_ft@yahoo.com.br (A.C.M.R.); 2 Laboratory of Marine Invertebrates Cell Biology, Institute of Biosciences, Universidade de São Paulo (USP), São Paulo 05508-090, SP, Brazil; mcust@usp.br; 3 Institute of Marine Sciences, Universidade Federal de São Paulo (UNIFESP), Santos 11070-100, SP, Brazil; fabio.simoes@unifesp.br (F.R.S.); gabi_martinsaraujo@hotmail.com (G.M.A.); 4 Department of Biophysics, Universidade Federal de São Paulo (UNIFESP), São Paulo 04044-020, SP, Brazil; miranda.unifesp@gmail.com (A.M.); pelopes2@yahoo.com.br (F.A.); 5 National Dental Centre Singapore, 5 Second Hospital Avenue, Singapore 168938, Singapore; na.yu0909@gmail.com

**Keywords:** spongin-like collagen, extraction protocols, marine sponges, biomaterials

## Abstract

This study aimed to compare different protocols (Protocol 1: P1; Protocol 2: P2; Protocol 3: P3; Protocol 4: P4) for the extraction of spongin-like collagen (SC) from marine sponges. The SEM micrographs demonstrated a fibrillar structure for the extracts from *Chondrilla caribensis* and the nodular/particulate aggregates for *Aplysina fulva*. FTIR showed for all samples peaks similar to collagen for both species. For *C. caribensis*, the extracts obtained using P2, P3, and P4 protocols presented higher values of extraction yield, TPQ, and GAGs. P2 and P4 showed higher values of SC concentration and for antioxidant analysis. For *A. fulva*, P2, P3, and P4 provided a higher extraction yield besides an increase in the antioxidant assay. For both species, no difference was observed for Col quantification and TPQ analysis; also, higher values of GAGs were found using P2 and P4. Fibroblast proliferation observed for *C. caribensis* was lower for P1 on day 1 and for P2 and P3 on day 3 (for 50%) compared to the control group. There was a significant reduction in fibroblast cell proliferation for all *A. fulva* extracts evaluated. It can be concluded that protocols P2 and P4 were more efficient for extracting SC from *C. caribensis*.

## 1. Introduction

Collagen (Col) is known as the most abundant protein in the extracellular matrix and plays an essential role in keeping its integrity and structure [1,2]. It has been widely explored as a promising material for tissue engineering and regenerative medicine applications such as bone and skin grafts [3,4]. It is biocompatible, presents low immunogenicity, and is able to support the adhesion and differentiation of many cell types [5].

Col can be extracted from many different sources, but it is mainly derived from bovine or porcine skin and bones [6]. However, some issues are related to the use of this type of Col, such as zoonosis transmission, potential immunogenic reactions, high costs, as well as ethical and religious concerns [7,8,9].

Biotechnology strategies have been developed in an attempt to overcome these limitations, primarily through the exploration of innovative sources of Col, including the natural biodiversity [10]. Natural bioactive compounds are often more biocompatible and offer a more efficient biological interaction for stimulation of tissue growth and repair [11,12]. In this context, marine life provides plentiful resource for the development of novel medical products [11,12]. Among them, the marine sponges (phylum Porifera) are one of the most promising sources of biological elements and molecules, with a vast potential for a wide range of applications mainly due to the antitumor, antiviral, and anti-inflammatory effects of their biocompounds [3].

Marine sponges are sessile animals considered representatives of the first multicellular animals [13]. In their structure and composition, there are many bioactive components [14,15], including marine Col [also known as spongin (SPG) or spongin-like collagen (SC)] [12,16]. SC has a similar composition to vertebrate Col and it has been considered as a natural compound for tissue bioregeneration, working as a cell–matrix adhesion framework [3,17]. In addition, SC is biocompatible and capable of supporting human skin cell growth [2,18]. Recently, [19] developed and compared four different methods of SC extraction from *Chondrosia reniformis Nardo* marine sponges. From the obtained extracts, two were used to manufacture a collagenous membrane, and its ability to support fibroblast and keratinocyte cell proliferation through in vitro studies was demonstrated.

However, there is a continuous need to develop optimized protocols for SC extraction of marine sponges, focusing on obtaining a faster, inexpensive and more efficient process [19]. Moreover, the use of “green” chemical products and processes to reduce or eliminate the use of hazardous substances in order to obtain natural products is also in high demand [9,19,20]. In this context, the present work focused on testing four different protocols for SC extraction from two marine sponge species (*Chondrilla caribensis* and *Aplysina fulva*); one of them served as a “control” once it has been used as a *standard* protocol in the literature [21]. Additionally, some amendments in the reagents (quantity/concentration) were performed for the other protocols (for example, the inclusion of trypsin, Tris-HCl buffer and deionized water) for more sustainable products, in order to try to obtain an optimized process. Moreover, in the present study a new technique, the cryogenic grinding, was first used for processing SC extracts of the marine sponges. The morphological and physicochemical characteristics of the extracts were obtained by scanning electron microscopy (SEM), Fourier transform infrared spectroscopy (FTIR), circular dichroism spectroscopy (CD), extraction yield quantification, SC, total protein quantification (TPQ), and glycosaminoglycans (GAGs) assay, as well as antioxidant activity evaluation. The in vitro biological effects were determined by measuring fibroblast cell proliferation.

## 2. Materials and Methods

### 2.1. Spongin-Like Collagen Extraction

The specimens of marine sponges *C. caribensis* and *A. fulva* were used in this study for SC extraction. Samples were collected after registration in the Brazilian National System for the Management of Genetic Heritage and Associated Traditional Knowledge (SISGEN), from coasts with high hydrodynamism in Praia Grande (23°49′23.76″ S, 45°25′01.79″ W, São Sebastião, Brazil) and in the intertidal zone in Ilha dos Papagaios, (22°53′51.4” S 41°58′56.1″ W, Cabo Frio, Brazil). The samples were immediately washed with seawater, placed in containers containing seawater and transported to the laboratory in thermal boxes. Three washing steps were performed in distilled water to remove cell debris, and samples were stored at −20 °C.

All samples were subjected to a pretreatment procedure for removing excess pigments and residues. It consisted of cutting 25 g of frozen sponge tissue into small pieces using a scalpel blade and placing the samples in a stirred beaker with distilled water for 2 h. Afterwards, the samples were frozen and freeze-dried before undergoing SC extraction protocols as described.

Protocol 1 (P1) was based on the work of [21]. For the extraction of SC, the marine sponge frozen samples were placed in a beaker containing 100 mM Tris-HCl buffer (10 mM EDTA, 8 M urea, 100 mM 2-mercaptoethanol, pH 9.5) and the pH was adjusted to 9 with the use of NaOH solution. Then, the obtained solution was transferred into a beaker and stirred for 24 h at room temperature. The solution was centrifuged (5000 rpm; 5 min. and 2 °C) (HETTICH ROTINA^®^ 420R, Tuttlingen, Germany). The pellet was discarded, and the supernatant was removed for analysis. The pH was adjusted again to 4 with the use of acetic acid and subjected to a new centrifugation step. At this stage, it was possible to observe formation of the precipitate. This precipitate was resuspended in distilled water and centrifuged again. The solution was freeze-dried for preservation of the SC [21].

For protocols 2 (P2), 3 (P3), and 4 (P4), the pretreated samples were ground into a fine powder using cryogenic milling (Retsch Mixer Mill, MM400, Haan, Germany) as described by [22] with some modifications. Briefly, the lyophilized marine sponge samples were placed in a 25 mL stainless steel grinding jar and subjected to pre-cooling at 30 Hz for 5 min with liquid nitrogen. The cryogenic grinding was performed for three cycles at 30 Hz for 2 min followed by cooling in liquid nitrogen. After the grinding process, the samples were stored at room temperature.

**Figure 1:**
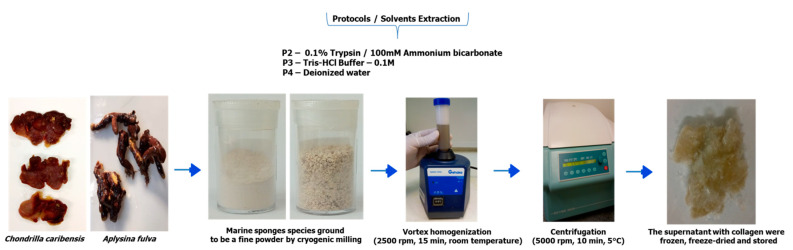
Summary of SC extraction methodology for *C. caribensis* and *A. fulva* for protocols 2, 3, and 4.

The obtained powder was well solubilized with the respective solvents (0.1% trypsin/100 mM ammonium bicarbonate (pH 8.5) for P2, 0.1M Tris-HCl buffer (pH 7.5) for P3, and deionized water (pH 6.8) for P4, in 50 mL Falcon tubes, using a vortex-mixer (AV-2, GEHAKA^®^, São Paulo, Brazil)) at 2500 rpm for 15 min at room temperature. The obtained powder was well solubilized with five volumes of the respective solvents (Figure 1). The mixture was then centrifuged (5000 rpm, 10 min at 5 °C). The supernatant was collected, dialyzed against deionized water (P2 and P3) (ratio 1:20), frozen at −20 °C, freeze-dried, and stored.

### 2.2. Material Characterization

#### 2.2.1. Scanning Electron Microscopy

The samples were mounted on aluminum stubs using carbon tape, sputter-coated with gold/palladium (System BAL-TEC MED 020, BAL-TEC, Balzers, Liechtenstein), and examined by SEM using a ZEISS LEO 440 microscope (20 kV, 2.82 A).

#### 2.2.2. Fourier Transform Infrared Spectroscopy

An FT-IR Spectrum Two spectrometer (Perkin, São Paulo, Brazil) was used to depict the chemical groups present in the SC by FTIR technique. The small amounts of SC in the powder form were homogenized with potassium bromide (KBr) pellets. The FTIR measurements were done in the range of 400–4000 cm^−1^ with a resolution of 2 cm^−1^. The samples were scanned 100 times for each FTIR measurement, and the spectrum acquired was an average of these scans.

#### 2.2.3. Circular Dichroism

CD spectroscopy was applied to assess alterations in the secondary structure of the Col molecule in the marine sponge extracts. Spectra of extracts in the concentration of 1 mg/mL were recorded with a J-815-JASCO^®^ spectropolarimeter (JASCO^®^, São Paulo, Brazil) in 0.5 mm path length quartz cuvettes from 190–250 nm at 25 °C, with a scan speed of 50 nm/s. The final spectra obtained was the average of four consecutive measurements. Distilled water was used as a blank.

#### 2.2.4. Extraction Yield Analysis

The extraction yield was expressed as the ratio of the dry extract obtained, per weight of the initial dry sample. Briefly, after the pre-treatment, 10 g of dry marine sponges were weighted (AUW, SHIMADZU, São Paulo, Brazil) and underwent specific extraction procedures (P1 to P4). The obtained extracts were freeze-dried for 24 h and measured again. The process was carried out in triplicate. Equation (1) was used to calculate the extraction yield for each protocol.

*Extraction yield* (%) = *Me*⁄*M* × 100 (1)

○ *Me* = mass of lyophilized extract in grams
○ *M* = mass of dry samples in grams after the pretreatment

#### 2.2.5. Spongin-Like Collagen Quantification

The modified method based on the Chloramine T reaction, determined by the estimation of the hydroxyproline content, was used for measuring the total SC for each extract [19]. Samples collected from each protocol (0.2 mL) were hydrolyzed with NaOH using an autoclave at 120 °C, for 20 min. After that, samples were neutralized by HCl and diluted in deionized water. The concentration of hydroxyproline was obtained by adding Chloramine T and Ehrlich’s reagent. Absorbance was measured at 550 nm using a Beckman spectrophotometer (DU 640) and compared to a cis-4-hydroxy-L-proline standard curve. Finally, the content of hydroxylated proline residue (Hyp) was used to infer SC content of each extract from the following formula:

SC content = 1 g Hyp × 10 g SC

The SC content was calculated using a proportion factor 1:10 of Hyp per collagen [23]. The procedure was carried out in duplicate.

#### 2.2.6. Total Protein Quantification

For TPQ analyses, the chosen method was the method used in [24]. Briefly, 5 mL of Coomassie Brilliant Blue BG-250 staining was added to 100 μL samples of P1, P2, P3, and P4. Samples were mixed and incubated at room temperature for 10 min. The absorbance of each sample was measured at 595 nm using a Beckman spectrophotometer (DU 640) and plotted against a bovine serum albumin straight-line equation (Y = 0.0089x + 0.0131). The procedure was carried out in duplicate.

#### 2.2.7. Alcian Blue Glycosaminoglycan Assay

The GAG content was measured for samples extracted by the experimental protocols using the Alcian blue GAG assay [25]. To 20 µL of each sample, 0.027 M H_2_SO_4_, 0.375% Triton X-100, and 4 M guanidine-HCl in a ratio of 1:1 (*v*/*v*) was added. GAGs were stained with 0.2 mL of working dye solution containing 0.25% Triton X-100, 0.018M H2SO4, and 0.005% Alcian blue stain. The samples were stirred for 10 min and centrifuged (16,000 rpm, 10 min at 4 °C). The supernatant was discarded, and the stained GAG pellet was mixed with 500 μL of 8 M guanidine-HCl solution. The absorbance of each sample was measured at 600 nm using a Beckman spectrophotometer (DU 640) and compared with a shark cartilage chondroitin sulfate standard curve. The procedure was carried out in duplicate.

#### 2.2.8. Antioxidant Activity Assay

The antioxidant activity in the samples extracted from marine sponge species was evaluated using the ABTS·+ method proposed by [26]. Briefly, 30 μL of each extract was added into 3 mL of 7 mM diluted ABTS·+ solution, homogenized, and sheltered from light. After 6 min, the absorbance was measured at 734 nm using a Beckman spectrophotometer (DU 640). The results were expressed as µmol of Trolox equivalent/g of extract and plotted against a Trolox standard curve of known concentration. The procedure was carried out in duplicate.

### 2.3. Cell Culture Studies

Cytotoxicity of SC and its influence on cell proliferation were assessed by an indirect assay [27] using extracts of the materials. The materials were sterilized using ultraviolet irradiation (UV) for 24 h. SC was maintained in standard α-MEM culture medium (Alpha Minimal Essential Medium with 10% fetal bovine serum and 1% antibiotic; Vitrocell, Campinas, Brazil) supplemented with 1% β-glycerophosphate, 1% 2-phospho-L-ascorbic acid trisodium salt, and 0.1% dexamethasone for 24 h in a humidified incubator set at 37 °C and 5% CO_2_. After this period, the culture medium containing the extracts were filtered using a 0.22 µm filter (KASVI^®^, Curitiba, Brazil). Control without material was incubated under the same conditions described above. In this study, murine fibroblasts cells (L929) [ATCC CCL-1; Bank of Cells of Rio de Janeiro (Banco de Células do Rio de Janeiro), BCRJ, RJ, Brazil] were used. L929 cells (BCRJ, RJ, Brazil) were cultured in a standard culture medium using a humidified incubator set at 37 °C and 5% CO_2_. Upon 80% confluency, cells were detached using trypsin and seeded at a density of 5 × 103 cells/cm^2^ in 24-well plates containing 1 mL of supplemented standard medium per well. After 24 h, the medium was substituted for 1 mL of composite extracts which were previously collected, and the cells were incubated for 1 and 3 days. Afterward, MTT assay (Thermo Fisher Scientific, São Paulo, Brazil) was performed on all samples, at each time point, in order to evaluate the cell viability. At the end of each trial period, the well plates were washed with phosphate buffered saline (PBS) and then MTT solution (50 μL, 0.5 mg/mL) was added into each well and incubated in the dark for 3 h. Then, 100 µL of isopropanol was aliquoted into wells of a 96-well plate to dissolve the formazan crystals. Absorbance values were measured in the microplate spectrophotometer (BioTek Instruments, Inc., Winooski, VT, USA) at 620 nm. From the values obtained, proliferation rates were calculated as the percentage reduction of MTT to formazan crystals.

### 2.4. Statistical Analysis

The obtained results were expressed as mean ± standard deviation (SD). The Shapiro–Wilk W test was used to verify the distribution of all variables. For the normally distributed variables, comparisons among extraction protocols were made with one-way analysis of variance (ANOVA) with post hoc Tukey multiple comparisons tests. In the case of non-normally distributed variables, the Kruskal–Wallis test was used. GraphPad Prism version 8.1.0 was used for the analyses. Statistical differences were considered significant at *p* ≤ 0.05 and signalized on graphics with an asterisk.

## 3. Results

### 3.1. Scanning Electron Microscopy Analysis

**Figure 2:**
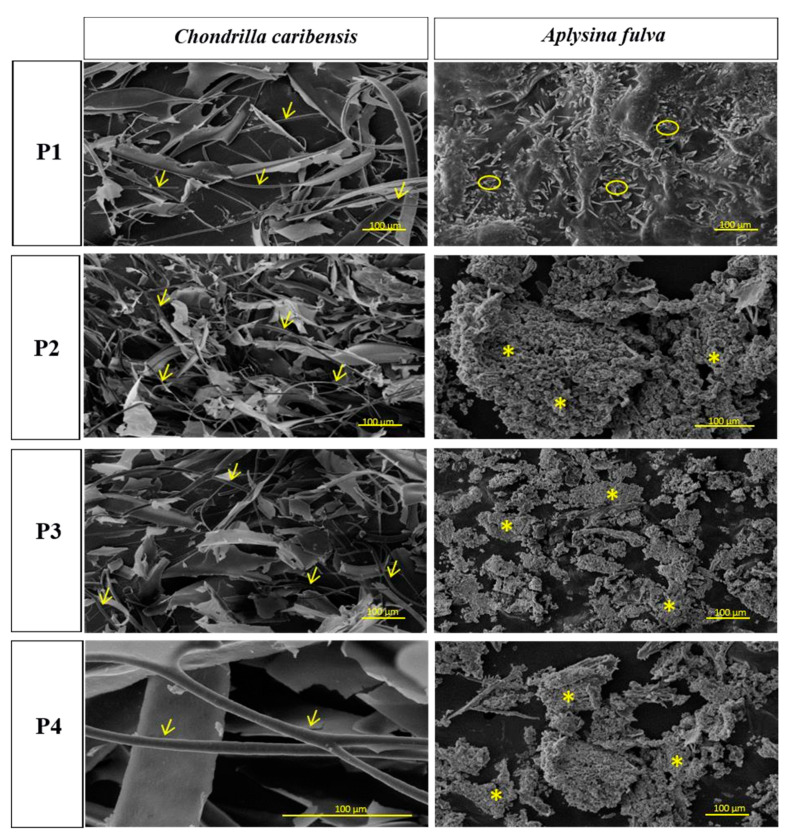
SEM micrographs of freeze-dried crude extracts. It is possible to observe fibrillar SC in all *C. caribensis* extracts (→). For *A. fulva*, a nodular/particulate material was depicted in P2, P3, and P4 (*) an P1(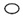) extracts, respectively.

Figure 2 represents the SEM micrographs of the extracted samples from *C. caribensis* and *A. fulva*. The images show a fibrillar SC structure cluttered with a tangle of fibers for *C. caribensis*. When comparing the extracts from the protocols used for this species, the images showed a periodic pattern. Comparable nodular aggregates were found in the P2, P3, and P4 extracts for *A. fulva*, while particulate matter was noticeable in the P1 protocol extract.

### 3.2. Fourier Transform Infrared Spectroscopy Investigation

**Figure 3:**
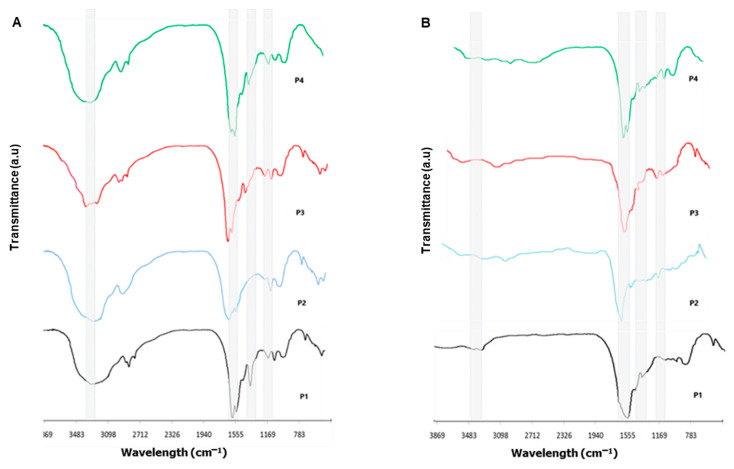
Organic compounds demonstrated by Fourier transform infrared spectra (FTIR) of extracts from marine sponge species (**A**) *C. caribensis* (**B**) *A. fulva*.

Figure 3A,B show the FTIR transmittance for all extracts of *C. caribensis* and *A. fulva* respectively. For *C. caribensis*, it was possible to observe bands attributed to N-H stretching vibration at 3273, 3297, 3228, and 3261 cm^−1^ for P1, P2, P3, and P4 respectively. Bands corresponding to stretching vibrations from the carbonyl groups (C=O bond) were noted at 1651, 1685, 1643, and 1639 cm^−1^ for P1, P2, P3, and P4 respectively. The bands corresponding to bending vibration from the N-H bond combined with stretching vibration from the C-N bond was observed at 1522, 1524, and 1532 cm^−1^ for P1, P3, and P4 respectively. FTIR transmittance spectra also showed bands at 1242, 1248, 1247, and 1239 cm^−1^ respectively, characteristic for amide III, in all tested protocols extracts (Figure 3A).

The *A. fulva* FTIR transmittance spectra demonstrated similarity to the bands obtained from *C. caribensis* extracts despite showing less prominent intensity peaks. Bands attributed to N-H stretching vibration can be observed at 3226, 3247, 3261, and 3234 cm^−1^ for P1, P2, P3, and P4 respectively. Carbonyl groups (C=O bond) were noted at 1634, 1629, 1637, and 1622cm^−1^ for P1, P2, P3, and P4 respectively. Bands depicting amide II were found at 1517, 1586, 1514, and 1522 cm^−1^, whereas amide III bands were registered at 1221, 1219, 1227, and 1229 cm^−1^ for all tested protocol extracts (P1, P2, P3, and P4 respectively) (Figure 3B).

### 3.3. Circular Dichroism Analysis

**Figure 4:**
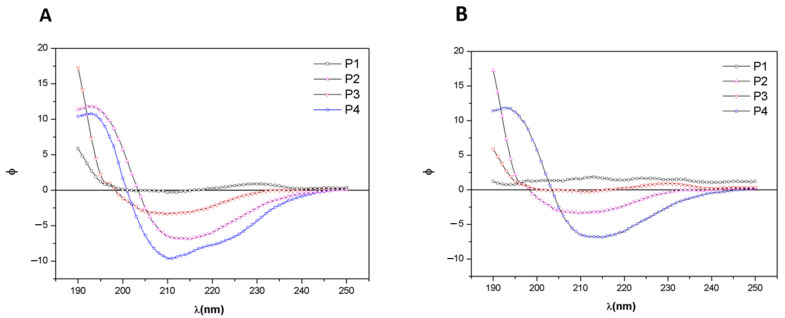
Circular dichroism (CD) spectra of extracts obtained from protocols P1, P2, P3, and P4 of the species (**A**) *C. caribensis* (**B**) *A. fulva*. The data are represented as dichroic ellipticity ([Θ]) versus wavelength.

The CD spectra of extracts from *C. caribensis* and *A. fulva* species are shown in Figure 4A,B respectively. For *C. caribensis*, CD spectra of P2 and P4 extracts presented a negative peak at 210 nm at 25 °C. On the other hand, a negative peak was present close to 212 nm for the *A. fulva* P4 extract.

### 3.4. Extraction Yield Analysis

**Figure 5:**
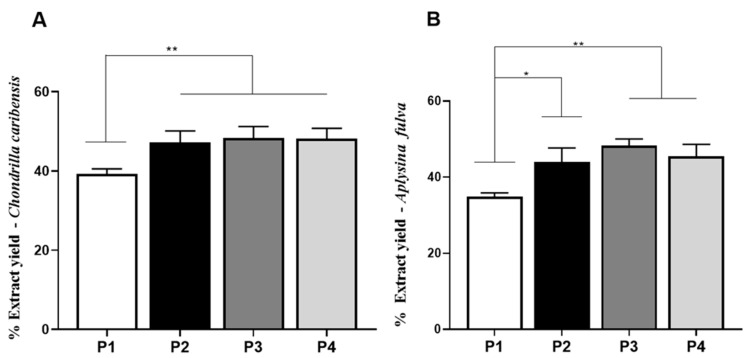
Extract yield evaluation. (**A**) Percentage of extract yield obtained from the specie *C. caribensis* using P1, P2, P3, and P4. Statistical differences were found between P1 vs. P2, P3 and P4 ** (*p* < 0.01). (**B**) Percentage of extract for *A. fulva* species using P1, P2, P3 and P4. Statistical differences were verified between P1 vs. P2 * (*p* = 0.0128) and P1 vs. P3 and P4 ** (*p* < 0.01). There was no statistical difference between protocols P2, P3 and P4 (*p* > 0.05). All analyzes were performed in triplicate (Anova/Tukey test).

Figure 5A,B demonstrated the percentage of extraction yield obtained with the four protocols for both marine sponge species. For *C. caribensis*, P2 (47.2 ± 2.8%, *p* = 0.0095), P3 (48.2 ± 2.5%, *p* = 0.0097), and P4 (48.3 ± 2.9%, *p* = 0.0098) had statistically significant higher values of extract yield compared to P1 (39.2 ± 1.3%). No other statistically significant difference was observed.

Similarly, for *A. fulva*, the extract yield of P2 (43.9 ± 3.7%, *p* = 0.0128), P3 (48.3 ± 1.7%, *p* = 0.0011), and P4 (45.4 ± 3.2%, *p* = 0.0053) were significantly higher compared to P1 (34.8 ± 1.1%). No other statistically significant difference was observed.

### 3.5. Spongin-Like Collagen Quantification

**Figure 6:**
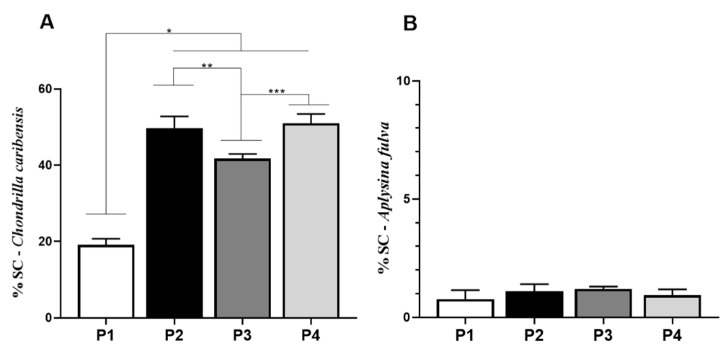
Percentage of SC obtained in extracts analysis by Cloramine T reaction method. (**A**) For the specie *C. caribensis*, between the protocols P1 vs. P2, P3 and P4 there were statistically significant differences * (*p* < 0.0001), P2 vs. P3 ** (*p* = 0.0121) and P3 vs. P4 *** (*p* = 0.0049). No statistically significant difference was found between protocol P2 vs. P4 (*p* > 0.05). (**B**) In contrast, for the specie *A. fulva*, no statistically significant differences were found for the tested protocols (*p* > 0.05). All analyses were performed in triplicate (Anova/Tukey test).

The SC quantification for *C. caribensis* is shown in Figure 6A. Univariate analysis revealed that the values for P1 (19.1 ± 1.6%) were statistically significantly lower compared to those for P2 (49.8 ± 3.0%, *p* = 0.0001), P3 (41.7 ± 1.2%, *p* = 0.0121), and P4 (51.0 ± 2.4%, *p* = 0.0049). Similarly, P3 demonstrated statistically significant lower values compared to P2 and P4. No other statistically significant difference was observed. *p* values were P1 vs. P2, P3 and P4 (*p* < 0.0001), P2 vs. P3 (*p* = 0.0121), and P3 vs. P4 (*p* = 0.0049). For Col quantification for *A. fulva*, no statistically significant difference was observed among the experimental groups (mean values of 0.7 ± 0.3%, 1.1 ± 0.3%, 1.1 ± 0.3%, 1.2 ± 0.1%, and 0.9 ± 0.2% for P1, P2, P3, and P4, respectively) (Figure 6B).

### 3.6. Total Protein Quantification

**Figure 7:**
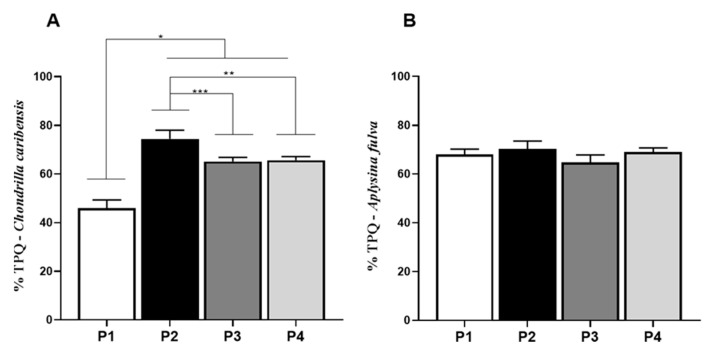
Percentage of total soluble proteins (TPQ). (**A**) Means and standard deviations of the percentage of TPQ in the extracts obtained (P1, P2, P3, and P4) for the specie *C. caribensis*. The highest protein solubilization was observed in P2 and the lowest in P1. Statistical differences were observed between the protocols P1 vs. P2, P3 and P4 * (*p* < 0.0001), between P2 vs. P3 *** (*p* = 0.0133) and P2 vs. P4 ** (*p* = 0.0179). No statistical differences were found between P3 vs. P4 (*p* > 0.05). (**B**) For *A. fulva* species, no statistical differences were found between the results for total protein extraction by the [24] method (*p* > 0.05). All analyses were performed in triplicate (Anova/Tukey test).

For TPQ, the average value for P1 (45.9 ± 3.4%) was significantly lower compared to the other groups (P2 = 74.5 ± 3.5%, P3 = 65.1 ± 1.7%, and P4 = 65.6 ± 1.5%, *p* < 0.0001) for *C. caribensis*. Further, values for P2 were statistically higher compared to P3 and P4 (74.5 ± 3.5%, (*p* = 0.0133). No other statistically significant difference was observed (Figure 7A). For the *A. fulva* extracts, no statistically significant difference was observed among the extraction protocols P1 (67.9 ± 2.2%), P2 (70.1 ± 3.3%), P3 (64.8 ± 3.1%), and P4 (69.1 ± 1.6%) (Figure 7B).

### 3.7. Alcian Blue Glycosaminoglycan Assay

**Figure 8:**
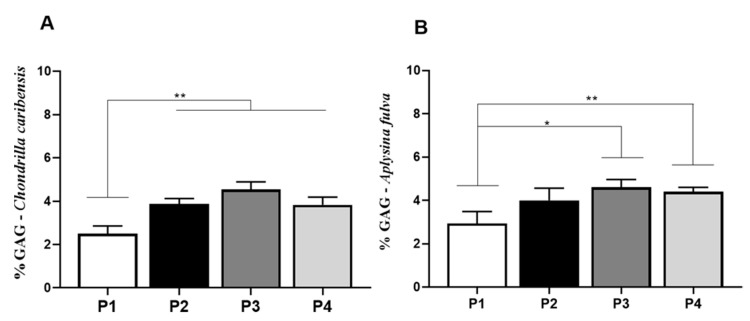
Percentage of GAG per extract obtained for the species *C. caribensis* and *A. fulva*. (**A**) C. caribensis extraction protocols P1, P2, P3, and P4, statistical differences were found between groups P1 vs. P2, P3 and P4 ** (*p* < 0.01). (**B**) For *A. fulva* specie for extraction protocols P1, P2, P3, and P4, statistical differences were found between groups P1 vs. P3, * (*p* = 0.0075) and P1 vs. P4 ** (*p* = 0.0154). All analyses were performed in triplicate (Anova/Tukey test).

For *C. caribensis*, univariate analysis demonstrated statistically significant lower percentage of GAGs for P1 (2.5 ± 0.3%) compared to P2 (3.8 ± 0.2%, *p* = 0.0044), P3 (4.5 ± 0.3%, *p* = 0.0003), and P4 (3.8 ± 0.3%, *p* = 0.0051). No other statistically significant difference was observed (Figure 8A). For *A. fulva*, GAG values for P1 (2.9 ± 0.5%, *p* = 0.0075) were significantly lower compared to P3 (4.6 ± 0.4%, *p* = 0.0154) (Figure 8B).

### 3.8. Antioxidant Activity Assay

**Figure 9:**
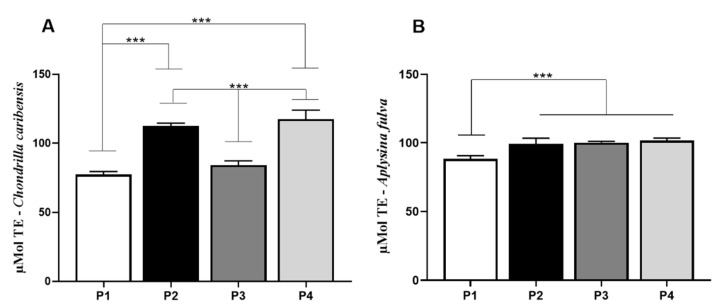
Means and standard deviations of the concentration in µMol TE/g extract for P1, P2, P3, and P4 extracts for *C. caribensis* and *A. fulva* species. (**A**) for the species, the highest antioxidant activity was observed between protocols P2 and P4, which did not present statistically significant differences between them. Statistical differences were observed between P1 vs. P2, P1 vs. P4, P2 vs. P3, P3 vs. P4 *** (*p* < 0.0001). (**B**) Means and standard deviations of the concentration in µMol TE/g extract for extracts P1, P2, P3, and P4 for *A. fulva* species, showed statistical differences between P1 vs. P2, P3 and P4 *** (*p* < 0.01). All analyses were performed in triplicate (Anova/Tukey test).

For *C. caribensis*, values found in the antioxidant assay for P1 (77.4 ± 2.1) were significantly lower compared to P2 (112.4 ± 2.2) and P4 (117.6 ± 6.4) (*p* < 0.0001). Similarly, P3 (84.1 ± 3.1) was significantly lower compared to P2 and P4. No other difference was observed (Figure 9A). For *A. fulva*, antioxidant assay demonstrated that P1 (88.2 ± 2.4) had lower values compared to P2 (99.17 ± 4.2), P3 (100.1 ± 0.9), and P4 (101.6 ± 1.8) (*p* < 0.0001) (Figure 9B).

### 3.9. Cell Culture Studies

**Figure 10:**
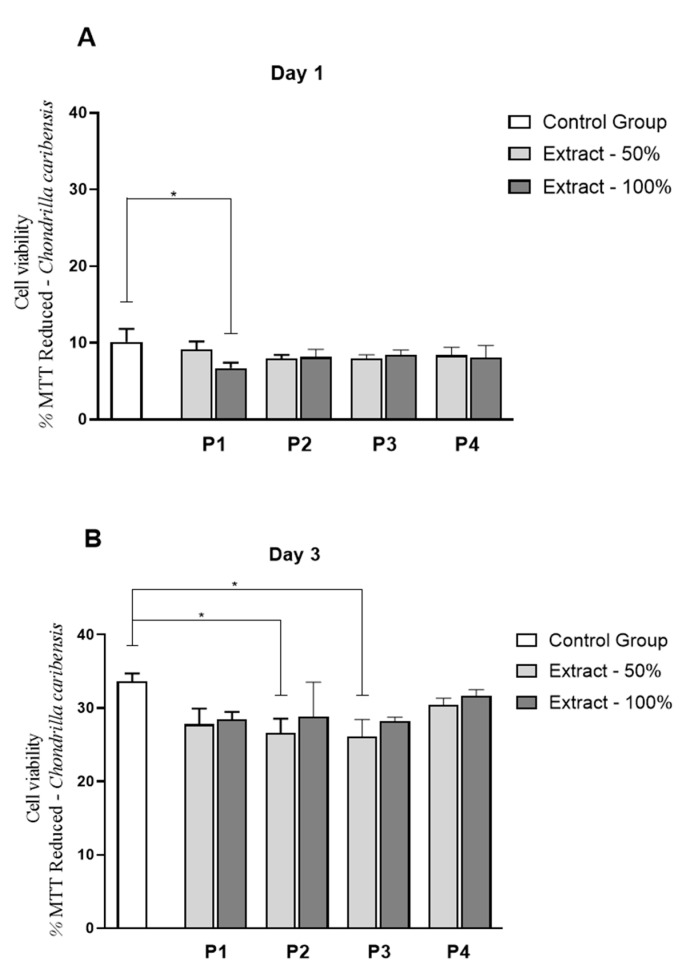
Viability L929 cell line by MTT assays in solution containing extracts of *C. caribensis* marine sponge species at experimental periods of 1 day (**A**) and 3 days (**B**). * *p* < 0.001 (Anova/Tukey test).

**Figure 11:**
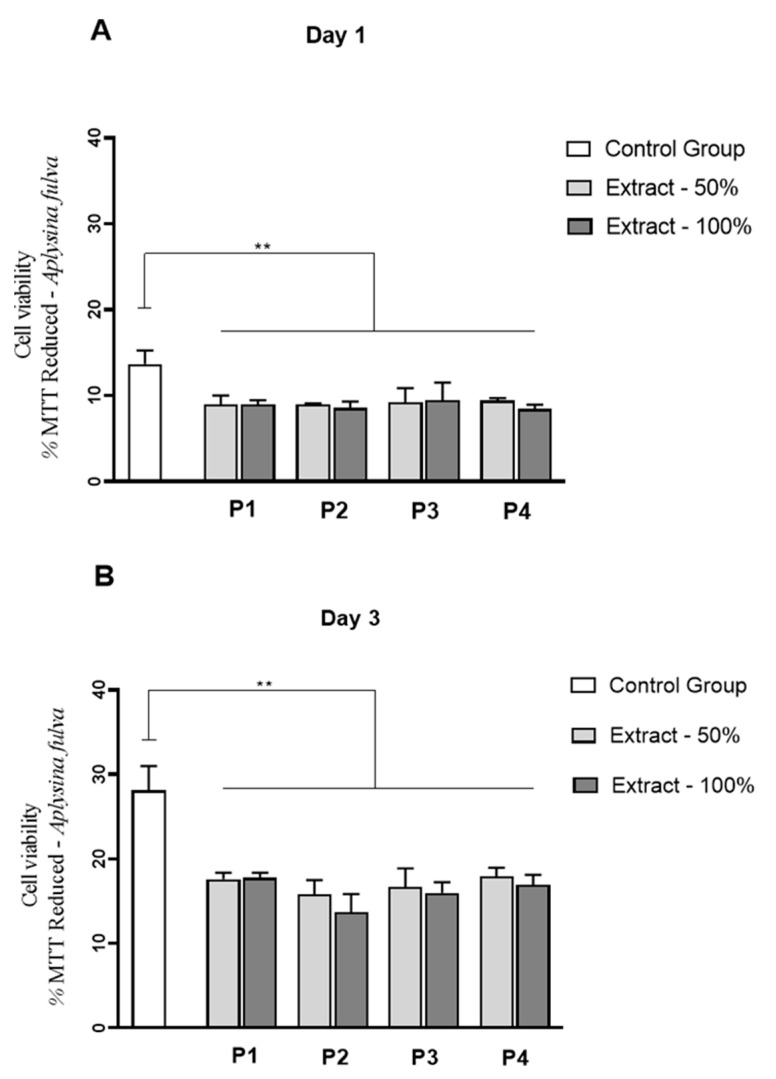
Viability L929 cell line by MTT assays for *A. fulva* protocols extracts at experimental periods of 1 day (**A**) and 3 days (**B**) of L929 culture. ** *p* < 0.0001 (Anova/Tukey test).

Figure 10 and Figure 11 demonstrate the values for the fibroblast cell proliferation for both marine sponge species. It can be observed that for *C. caribensis*, a statistically significant reduction was observed in P1 (extract 100%) on day 1 (*p* = 0.0296), compared to control (Figure 10A). On day 3, a statistically significant decrease was demonstrated for P2 and P3 cultured in extract concentration 50% compared to control (*p* = 0.0248 and *p* = 0.0130, respectively) (Figure 10B).

Interestingly, for A. fulva extracts, a significant reduction in fibroblast cell proliferation was observed for all the extracts, at 50 and 100% compared to control, on days 1 and 3 of culture (*p* < 0.0001) (Figure 11A,B). No statistically significant differences among the experimental groups were observed.

## 4. Discussion

The present work aimed to compare four protocols of spongin-like collagen extraction from *C. caribensis* and *A. fulva* marine sponges for developing a more efficient, faster, and green chemistry-based protocol. SEM micrographs demonstrated a fibrillar structure for the extracts from *C. caribensis* and nodular/particulate aggregates for *A. fulva*. FTIR showed that both species presented transmittance bands attributed to N-H and carbonyl groups, N-H bonds combined with C-N stretches, and amide III for all tested protocols. The CD spectra of extracts from *C. caribensis* showed a negative peak at 210 nm for P2 and P4 while for *A. fulva* extracts, a negative peak was present close to 212 nm for the P4 extract.

For *C. caribensis*, the extracts obtained using P2, P3, and P4 presented higher values of extraction yield, TPQ, and GAGs, while those from P2 and P4 presented higher concentration of Col and antioxidant activity. For *A. fulva*, P2, P3, and P4 protocols produced higher extraction yield and superior values for antioxidant activity compared to P1. However, for this species, no statistically significant differences were observed for Col quantification and TPQ although higher values of GAGs were found using P2 and P4. Fibroblast cell proliferation observed for *C. caribensis* was lower for P1 (for 100%) on day 1, and P2 and P3 on day 3 (for 50%) compared to the control group. Interestingly, for *A. fulva* extracts, a statistically significant reduction in fibroblast cell proliferation was observed for all the extracts (at 50 and 100% compared to control on days 1 and 3).

The fibrillary structure for *C. caribensis* extracts in the present study obtained by SEM indicate that the cryogenic milling process was capable of preserving the structure. Also observed by [28], using transmission electron microscopy analysis, a similar fibrillary aspect of a marine collagen extract of *Chondrosia reniformis* Nardo. The nodular morphology observed for *A. fulva* extracts in the present study was also found by [16].

FTIR analysis demonstrated similar patterns for all the extracts of both species, which suggest that their chemical compositions are relatively similar. FTIR spectra of the organic part of marine sponge extracts are very complex, with several bands related to Col groups and other proteins, lipids, carbohydrates, and nucleic acids [11]. Barros et al. (2015) found in the FTIR spectra of the organic part of *Chondrilla nuculla* species several bands representing amide A, amide B, amide I, amide II, and amide III. These findings were similar to those found for other marine sources reported in the literature [29,30].

CD evaluation demonstrated negative peaks for samples extracted using P4 for both species (approximately 210 nm). Barros et al. (2015) found negative peaks (192 nm) in the CD analysis of Col/gelatin from *Chondrilla nuculla*, indicating that it is compatible with the characteristic profile of the Col triple helix. Moreover, it seems that the negative peaks close to 192 nm are similar to the findings for Col [11].

Higher extraction yields were seen using P2, P3, and P4 for both species, demonstrating the optimized effects of using smaller particle samples (obtained with the use of the cryogenic milling). Moreover, these results showed the efficiency of the solvents used in these protocols, which could improve Col and other poorly soluble substances released from both species. Most of the protocols for SC extraction used in the literature are based on acetic acid solutions and resulted in low extraction yields of around 2% [11,31]. Interestingly, no difference was observed in the extraction yield using an acidic, basic, and close to neutral pH in our study (ranging from 6.8 to 8.5 pH). Thus, we hypothesize that the positive values found for this variable could be related to the reduced particles of the samples obtained by cryogenic milling. This is because it is well-known that smaller particle sizes result in increased superficial area, which could have culminated in an enhanced dissolution rate and increased material solubilization [32].

Barros et al. (2015) demonstrated a 54% improvement of the extraction yield for *Chondrilla nuculla* (Alassio) and 82% yield for *Chondrilla nuculla* (Portofino) sponges. Pozzolini et al. (2018) demonstrated a significant improvement in the extraction yield for *Chondrosia reniformis* using an extraction protocol based on 2-mercaptoethanol which produced higher sponge tissue disaggregation. Thus, the addition of the reducing agent is likely to act at the disulphide-bond level, promoting complete sponge tissue disruption in the F3 and F4 extraction procedures.

There were significantly higher Col amounts for *C. caribensis* extracted using P2 with an alkaline medium (pH 8.5) and P4 with slightly acidic conditions (pH 6.8). A higher amount of protein was also found using P2. These findings are in agreement with [19] who also observed that trypsin digestion at an optimum alkaline pH range resulted in a higher amount of Col. Barros et al. (2015) also successfully extracted Col/gelatin using water acidified with carbon dioxide. Interestingly, previous works have stated that Col from marine sponge was insoluble in dilute acid pH mediums [21,33]. However, this behavior was not found in the present study with the use of slightly acidic pH (P4), possibly due to the presentation of the samples (fine powder).

Interestingly, for *A. fulva* no differences were observed for Col and protein evaluation for any protocol used, possibly due to the composition of this species. It is worthwhile to state that although it is well-known that *A. fulva* sponges present an organic composition, the protocols for SC extraction have usually included significant amounts of uncharacterized and unspecific microscopic materials that are retained in the final extracts despite centrifugation [11,19] which may explain the results for this species.

For the GAG assay, *C. caribensis* demonstrated significantly higher values for P2, P3, and P4 compared to P1. For the antioxidant assay, significantly higher values were found for P2 and P4 compared to P1 and P3. The same pattern was observed for the *A. fulva* species, where the values for GAG evaluation of P3 and P4 were higher than P1, and for the antioxidant assay where P2, P3, and P4 demonstrated significantly higher values compared to P1. These results may be explained by the technique of Col extraction (pulverization), which improved dissolution of the material and allowed superior extraction of the GAG and antioxidant components. GAG extraction has previously been successful in *A. fulva* and *Chondrilla nuculla* [34] and *Chondrosia reniformis* [19] marine sponges.

Considering the results for *C. caribensis*, the values of fibroblast cell proliferation were similar for all extracts compared to the control group (with the exception of lower values for P1 100% on day 1; and P2 and P3, both 50% on day 3). However, it is essential to highlight that there was no reduction in cell proliferation of more than 20% compared to the control group. Following the ISO 109333-5:2009, a material to be used for medical proposals can be considered toxic for cells if it produces more than 30% of death. In this context, it can be stated that the extracts of SC from all protocols of *C. caribensis* presented non-cytotoxicity. Barros et al. (2015) found that Col extracted from *Chondrosia reniformis* and *Chondrilla nuculla* (Alassio and Portofino) increased the metabolic activity of fibroblasts. Pozzolini et al. (2018) also demonstrated that a membrane manufactured using Col from the species *Chondrosia reniformis* was able to sustain fibroblast and keratinocyte growth, showing biocompatibility. Conversely, for *A. fulva*, all the extracts presented reduced fibroblast cell proliferation compared to control (around 35% of reduction), suggesting that the species may show a toxic effect on this cell lineage. It is crucial to point out that this work was limited to a preliminary study to investigate the biocompatibility of the marine SC from both species, and new studies must be performed to investigate the biological behavior of the extracts further.

Many different works in the literature used protocols based on acidic solutions for extracting and solubilizing SC from different sources [11,35,36]. Most of them are based on diluting samples in acetic acid solutions but they result in low SC yield [11,35,36]. In this context, the protocols used in the present study investigated the use of alkaline, a neutral, and an acid protocol. In general, for *C. caribensis*, the slightly acidic pH in deionized water and the basic pH in the enzymatic treatment with trypsin presented the most optimized results of extraction. These findings seem to be extremely attractive options for SC extraction, especially from the perspectives of green and sustainable chemistry and in terms of time and operating costs.

Conversely, for *A. fulva*, the protocols used were not suitable for SC extraction mainly based on the Col and protein evaluation and in vitro biological results. Furthermore, the use of cryogenic milling for processing the samples is a new approach in terms of SC extraction from marine sponges. The use of smaller particles for SC extraction seems to have improved solubilization and increased Col release. Thus, trypsin and water-based protocols, associated with the use of cryogenic milling for obtaining small particles, are able to successfully extract SC from *C. caribensis*.

## 5. Conclusions

As a conclusion, the results of the present work indicate that P2 and P4 protocols culminated in improvement of nearly 50% in the amount of SC extracted. Moreover, the amount of Col, protein, GAGs, and antioxidant activity were also increased with the use of P2 and P4. Interestingly, fibroblast cell proliferation was also increased when cultured in the presence of *C. caribensis* extracts. For *A. fulva* extracts no positive results were found, especially considering the low amount of Col and proteins obtained and the lower cell proliferation for fibroblasts. It is worthwhile to emphasize that the use of trypsin and water-based protocols was more efficient than the other reagents for extracting SC from small particles of *C. caribensis*.

All of the data found in the present work suggest that improved protocols for SC extraction were obtained especially for *C. caribensis* specie. As a consequence, the SC obtained may constitute a promising biomaterial to be used for tissue engineering proposals.
